# Modified entropy-based procedure detects gene-gene-interactions in unconventional genetic models

**DOI:** 10.1186/s12920-020-0703-4

**Published:** 2020-04-23

**Authors:** Jörg Malten, Inke R. König

**Affiliations:** Institut für Medizinische Biometrie und Statistik, Universität zu Lübeck, Universitätsklinikum Schleswig-Holstein, Campus Lübeck, Ratzeburger Allee 160, 23562 Lübeck, Germany

**Keywords:** Gene-gene-interactions, Entropy, IGENT

## Abstract

**Background:**

Since it is assumed that genetic interactions play an important role in understanding the mechanisms of complex diseases, different statistical approaches have been suggested in recent years for this task. One interesting approach is the entropy-based IGENT method by Kwon et al. that promises an efficient detection of main effects and interaction effects simultaneously. However, a modification is required if the aim is to only detect interaction effects.

**Methods:**

Based on the IGENT method, we present a modification that leads to a conditional mutual information based approach under the condition of linkage equilibrium. The modified estimator is investigated in a comprehensive simulation based on five genetic interaction models and applied to real data from the genome-wide association study by the North American Rheumatoid Arthritis Consortium (NARAC).

**Results:**

The presented modification of IGENT controls the type I error in all simulated constellations. Furthermore, it provides high power for detecting pure interactions specifically on unconventional genetic models both in simulation and real data.

**Conclusions:**

The proposed method uses the IGENT software, which is free available, simple and fast, and detects pure interactions on unconventional genetic models. Our results demonstrate that this modification is an attractive complement to established analysis methods.

## Background

It is generally assumed that genetic interactions play an important role in understanding the mechanisms of complex diseases such as coronary heart disease, Alzheimer’s disease, breast cancer or diabetes [1]. In the statistical sense, interaction refers to a situation in which the effect of one factor depends on the values of another factor on a given scale. In our case, genetic interactions come in two flavours. Firstly, interactions between genetic loci, usually termed gene-gene interactions or epistasis, occur, as described for rheumatoid arthritis (RA) [2–4]. Specifically, Liu et al. [2] described interactions between the locations DQA2 and DQB2 in the HLA region on chromosome 6. Secondly, gene-environment interactions denote interactions between genetic and environmental factors [5]. For example, Chandra et al. reported that the interaction between serum cholesterol levels and the sigma4 genotype [6] plays a role in Alzheimer’s disease.

As a starting point, we will in this paper focus on the detection of gene-gene interactions in a case-control setting, although interactions of higher order can also be of interest. For simplification, our description is restricted to the situation where diallelic genetic markers such as single nucleotide polymorphisms (SNPs) are used leading to three possible genotypes. However, the results are generalizable to gene-environment-interactions with categorical environmental factors or indeed interactions of any categorical variables.

Generally, a single locus testing strategy is undertaken as the primary analysis in a genome-wide association study (GWAS), but this may be unsuitable to detect loci that interact with other variants since the relevant loci may not display effects on their own [7]. A variety of methods exists to detect or control for the presence of gene-gene interactions [8]. For a binary phenotype, most of them are based on the saturated logistic regression model for interaction [9, 10] or a simplication of it. The regression parameters are penetrances, odds or log odds. As Cordell [8] notes, this procedure implicitly assumes that the scale used for the regression parameters is the scale of interest. The saturated model has nine two-locus genotypes that are modeled by one intercept, four main effects parameters and four interaction parameters with a dummy coding of the genotypes. Although the saturated model is the best fitting one, a model with fewer parameters might be preferable, e.g. because of greater stability. This can be achieved by assuming a specific genetic model and thus estimating, for example, the additive effect of the number of risk alleles at both genetic loci. In this case, only one parameter is estimated for the interaction effect. In this line, the standard software PLINK [11] provides an overall 4 degree-of-freedom (df) test for interaction or a derived 1-df test assuming additive effects at both loci.

However, interaction models have been observed in reality that cannot easily be described by regression models for gene-gene-interactions without dummy coding. An example is given by Ziegler and König for sporadic breast cancer [10]. Therefore, the restriction to linear models is not optimal. As an alternative, a number of novel techniques have been developed in the last years that are based on different concepts such as rank building (ANOVA technique) [12], data-mining with the multifactor dimensionality reduction (MDR) approach [13, 14], machine learning methods with random forests (RF) [14, 15] or with support vector machines (SVM) [16]. Although promising, it is not always clear which interaction effects can be reliably detected by these methods [17].

Another new idea is borrowed from information theory, the entropy-based method. This concept is model-free and measures the uncertainty or disorder in a system and could therefore lend itself to detect interactions for many genotype constellations. This technique is suggested as particularly powerful and, because of the nonlinearity, as better able to capture nonlinear relationships between genetic variants or other variables [18]. Ferrario et al. reviewed different entropy-based measures providing information on suggested test statistics, simulations and implementations [18]. Focusing on second order interaction, there are three important concepts, namely conditional mutual information, information gain, and relative information gain. These are based on the following definitions: First, entropy can be defined as a measure for uncertainty in a random variable [19]. Then, mutual information refers to the reduction of uncertainty of one variable conditional on the knowledge of the other variable [18]. Furthermore, mutual information can also be conditioned on a third variable yielding the conditional mutual information (CMI) [18], which has been used for a test statistic by Zuo et al. [20]. Second, the term information gain is defined in different ways: Fan et al. [21] subtract the mutual information of two genetic variants estimated in the cases from the same quantities estimated in the controls. Alternatively, in the method IGENT Kwon et al. [22] subtract the conditional entropy of the phenotype, given two genetic variants, from the entropy of the phenotype. Kwon et al. [22] also work with the so-called relative information gain, which is given as the relation between the information gain and the entropy of the phenotype.

The advantage of the approach by Kwon et al. lies in the freely available and fast implementation that is also called IGENT [18]. The software has been implemented in C++ and is available at http://statgen.snu.ac.kr/software/igent/(different from the information in the paper from 2014 [22]). In contrast, Zuo et al. [20] work with an individual software.

One characteristic of entropy-based procedures is that main effects may also present themselves as a deviation from disorder, i.e. the entropy falls and the test reacts. This is an advantage if we search for main effects and interaction effects simultaneously and do not need to distinguish between either. This might be the case in a first step of an analysis in which the focus is on generally learning about variants having an effect on the outcome. Also, this offers the possibility to reduce the variants for a second computationally more intensive step. However, it should be noted that if we are interested in interaction effects only, a main effect of one or both genetic variants without an interaction will lead to a false positive result. Zuo et al. [20] state that the CMI concept achieves better or comparable control of the false positive error, compared to four previously proposed model-free metrics [20].

In the following, we are only interested in interaction effects, so it is necessary to eliminate main effects, without diminishing the advantages of the entropy approach. We therefore introduce a modification of IGENT that eliminates the problem of the increased type-I-error in the case of only main effects, but keeps the advantages of the entropy method as far as possible. We illustrate the behavior of the proposed procedure with data simulated for different genotypic models and apply it to the analysis of real data on the genetic background of RA [23, 24]). The same data set was analyzed previously by Liu et al. [2], who utilized a regression-based approach combined with random forest analyses and Chattopadhyay et al. [3] who worked with three non-parametric scores. Furthermore, most comparisons of interaction methods have so far focused on assessing deviation from additive or multiplicative effects. However, we assume that the strongest advantage of entropy-based methods is seen in more unconventional interaction models not following classical genetic models, and we considered these unconventional interaction models in our simulations.

## Methods

### Entropy and IGENT-estimator

Entropy is originally a term from thermodynamics referring to the level of disorder or uncertainty. Information theory has utilized this phenomenon as a measure for the lack of structure in a system [19]. Shannon defines the entropy H of a set of probabilities *p*_1_,…,*p*_*n*_ as $ - \sum p_{i} \log p_{i} $.

In the context of a disease state *D* depending on the genotypes at two genetic loci, Kwon et al. [22] derive the Information Gain similarly as follows:

First let entropy of the phenotype be written as

1 $$\begin{array}{@{}rcl@{}} H(D) &=& - \sum_{k=0}^{1} P(D_{k}) \log_{2} P(D_{k}) \end{array} $$

with *D*_0_ and *D*_1_ denoting the unaffected and affected state, respectively.

The second order entropy (conditional entropy of the disease state on the genotypes *G*) is then given by the expression

2 $$\begin{array}{@{}rcl@{}} H(D|G) &=& - \sum_{i,j=0}^{2} \sum_{k=0}^{1} P(G_{ij}) P(D_{k}|G_{ij}) \log_{2} P(D_{k}|G_{ij}) \\ &=& - \sum_{i,j=0}^{2} \sum_{k=0}^{1} P(G_{ij}, D_{k}) \log_{2} P(D_{k}|G_{ij}). \end{array} $$

Here, for (*i*,*j*=0,1,2), *i* and *j* define the genotype at the 1st and 2nd locus. Thus, all possible genetic models are considered.

From that, the Information Gain can be derived as

3 $$\begin{array}{@{}rcl@{}} IG(D|G) &=& H(D) - H(D|G) \\ &=& \sum_{i,j=0}^{2} \sum_{k=0}^{1} P(G_{ij}, D_{k}) \log_{2} \left(\frac{ P(G_{ij}, D_{k}) }{ P(G_{ij})P(D_{k})} \right). \end{array} $$

The estimator (2nd order) then leads to

4 $$\begin{array}{@{}rcl@{}} \widehat {IG}(D|G) &=& \sum_{i,j=0}^{2} \sum_{k=0}^{1} \hat P_{ijk} log_{2} \left(\frac{ \hat P_{ijk} }{ \hat P_{ij.} \hat P_{..k}} \right). \end{array} $$

For this, *X*_*ijk*_ are the observations of *G*_*ij*_ and *D*_*k*_ in *N* individuals, leading to the mean $\bar {X} = \sum _{i,j=0}^{2} \sum _{k=0}^{1} X_{ijk}$.

Then, $\hat P_{ijk} = X_{ijk} / \bar {X}$, $\hat P_{ij.} = \sum _{k=0}^{1} P_{ijk} $ and $\hat P_{..k} = \sum _{i,j=0}^{2} {P_{ijk}}$.

Furthermore we define the mutual information as

5 $$\begin{array}{@{}rcl@{}} MI(G) &=& \sum_{i,j=0}^{2} P(G_{ij}) \log_{2} \left(\frac{P(G_{ij}) }{P(G_{i.})P(G_{.j})} \right). \end{array} $$

Finally, the conditional mutual information is given by

6 $$ {{}\begin{aligned} CMI(G) = \sum_{i,j=0}^{2} \sum_{k=0}^{1} P(G_{ij}, D_{k}) \log_{2} \left (\frac{P(G_{ij}|D_{k}) }{P(G_{i.}|D_{k})P(G_{.j}|D_{k})} \right) \end{aligned}}  $$

Considering two genetic loci with three genotypes each, we construct 3×3 contingency tables which tabulate the values in terms of penetrances or odds for the resulting 9 genotype combinations. In this scenario, the IGENT method estimates an imbalance of the 9 odds, i.e., the deviation for the value of 1 for all odds.

The estimator of *IG*(*D*|*G*) (IGENT-estimator) asymptotically and approximately follows a gamma distribution under the null hypothesis that genotype combinations and disease states are independent. This null hypothesis is also violated in the case of association with one or both of the genetic variants. Thus, the type-I-error is inflated if this estimator is used as a test for interactions only.

### Modification of the iGENT-estimator

In the following, we want to utilize the IGENT approach while eliminating the influence of main effects so as to yield a purely interactive effect etimator. For this, we first estimate the main effect of one genetic variant by applying the IGENT approach to a 1st order calculation. Specifically, the Information Gain of 1st order for the first genotype is given by

7 $$\begin{array}{@{}rcl@{}} IG(D|G_{1})  &=& H(D) - H(D|G_{1}) \\ &=& \sum_{i=0}^{2} \sum_{k=0}^{1} P(G_{i.}, D_{k}) \log_{2} \left (\frac{P(G_{i.}, D_{k}) }{P(G_{i.})P(D_{k})} \right) \end{array} $$

This can be estimated by

8 $$\begin{array}{@{}rcl@{}} \widehat{IG}(D|G_{1}) &=& \sum_{i=0}^{2} \sum_{k=0}^{1} \hat P_{i.k} \log_{2} \left (\frac{ \hat P_{i.k} }{ \hat P_{i..}\hat P_{..k}} \right) \\ &=& \sum_{i,j=0}^{2} \sum_{k=0}^{1} \hat P_{ijk} \log_{2} \left (\frac{ \hat P_{i.k} }{ \hat P_{i..}\hat P_{..k}} \right) \end{array} $$

with $\hat P_{i.k} = \sum _{j=0}^{2} {P_{ijk}}$.

The estimator (1st order) for the second genotype is given analogously.

This leads to the following intuitive modification of IGENT:

9 $$\begin{array}{@{}rcl@{}} IGmod(D|G) &=& IG(D|G) - IG(D|G_{1}) - IG(D|G_{2}) \end{array} $$

All three components can be estimated within the IGENT software.

The estimator of *IGmod* is

10 $$\begin{array}{@{}rcl@{}} \widehat{IGmod}(D|G) &=& \widehat {IG}(D|G) - \widehat{IG}(D|G_{1}) - \widehat{IG}(D|G_{2}) \\ &=& \sum_{i,j=0}^{2} \sum_{k=0}^{1} \hat P_{ijk} \log_{2} \left (\frac{ \hat E_{ijk} }{ \hat L_{ij.}} \right) \end{array} $$

with $ \hat E_{ijk} = \frac { \hat P_{ijk} \hat P_{..k} }{ \hat P_{i.k} \hat P_{.jk} }$ and $ \hat L_{ij.} = \frac { \hat P_{ij.} }{ \hat P_{i..} \hat P_{.j.} }$.

Here, the factor $\hat E_{ijk}$ takes the value 1 if the two genotypes are conditionally independent, and the factor $\hat L_{ij.}$ equals 1 under the condition of no correlation between the genetic variants, i.e., no linkage disequilibrium (LD).

Under this condition of no LD, we can simplify the formula for $\widehat {IGmod}(D|G)$ to

11 $$\begin{array}{@{}rcl@{}} \widehat{IGmod}_{0}(D|G) &=& \sum_{k=0}^{1} \sum_{i,j=0}^{2} \hat P_{ijk} \log_{2} \left(\frac{\hat P^{k}_{ij}}{\hat P^{k}_{i.}\hat P^{k}_{.j}} \right) \end{array} $$

with $\hat P^{k}_{ij} = \frac {\hat P_{ijk}}{\hat P_{..k}}$, $\hat P^{k}_{i.} = \frac {\hat P_{i.k}}{\hat P_{..k}}$, and $\hat P^{k}_{.j} = \frac {\hat P_{.jk}}{\hat P_{..k}}$.

This conversion shows that this estimator works with conditional mutual information. Specifically, it estimates the deviation from the conditional independence, and it follows asymptotically and approximately a gamma distribution with shape-parameter 4 and scale parameter $\frac { 1}{N \ln (2)}$ under the null hypothesis of conditional independence of the genetic variants [25].

### Comparison with genoCMI

Recently, Zuo et al. [20] introduced an estimator called GenoCMI that was defined as follows:

12 $$ {\begin{aligned} GenoCMI &= \sum_{k=0}^{1} \sum_{i,j=0}^{2} P(G_{ij}, D_{k}) \ln\\&\quad \left (\frac{P(G_{ij} | D_{k})} {P(G_{i.}| D_{k}) P(P(G_{.j} | D_{k})} \right). \end{aligned}}  $$

Under the condition of no LD, this is identical to our modified IGENT-estimator $\widehat {IGmod}$, except for the basis of the logarithm. GenoCMI follows asymptotically and approximately a *χ*^2^(*ν*)/2*N* distribution where the degree of freedom *ν* is 8. This statement is equivalent to the above result of a gamma distribution with shape parameter 4 and scale parameter $\frac { 1}{N \ln (2)}$. An obvious disadvantage of GenoCMI is that there is no freely available software implementation, whereas IGENT is freely available and efficiently implemented.

### Simulation models for gene-gene-interactions

**Fig. 1:**
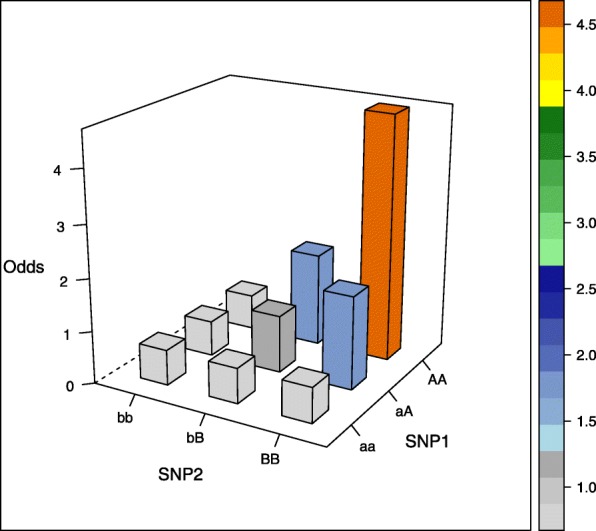
Odds in case-control scenario of model 1 (multiplicative model) with MAF=0.4

**Fig. 2:**
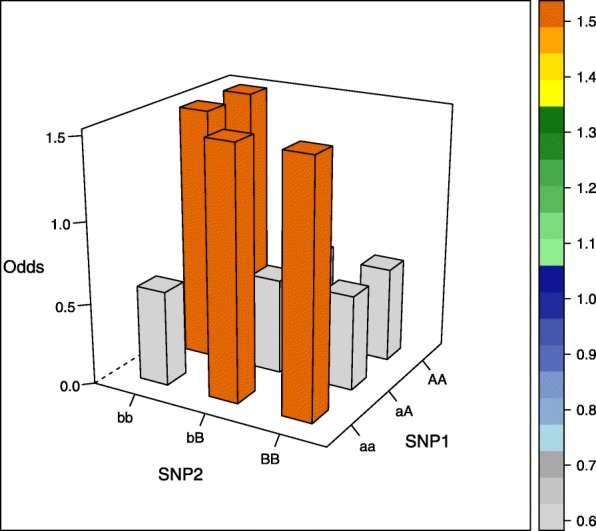
Odds in case-control scenario of model 2 (epistasis model) with MAF=0.4

**Fig. 3:**
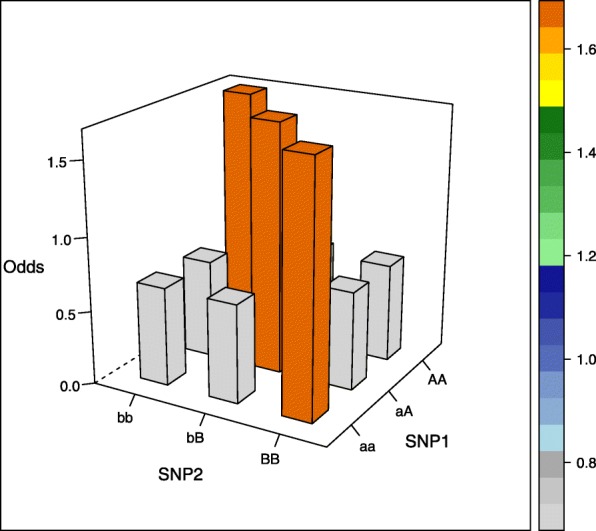
Odds in case-control scenario of model 3 (Two allele interaction model) with MAF=0.4

**Fig. 4:**
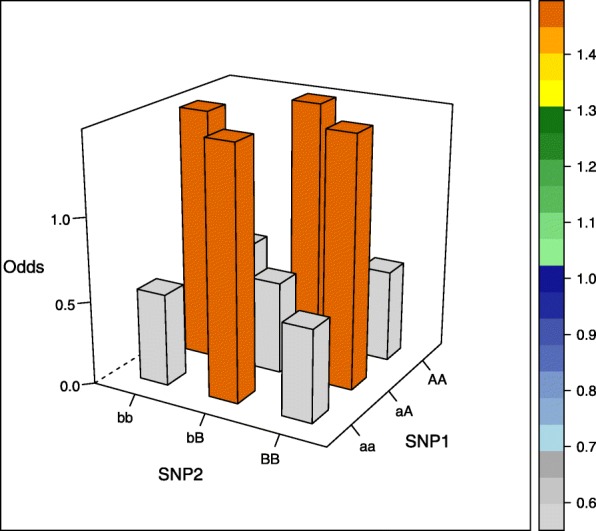
Odds in case-control scenario of model 4 (XOR model) with MAF=0.4

**Fig. 5:**
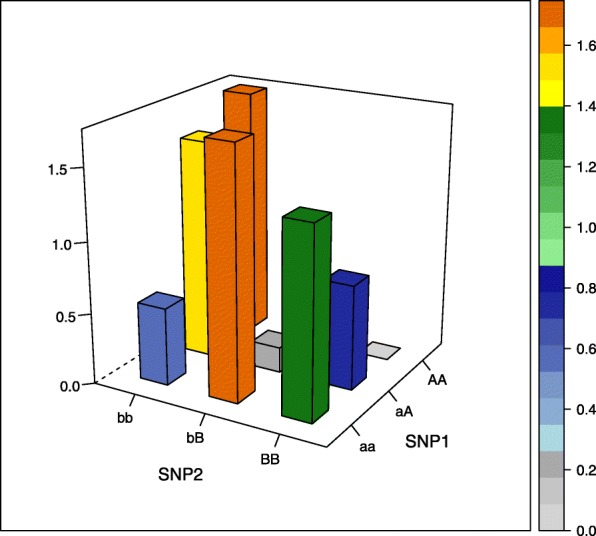
Odds in case-control scenario of model 5 (Model with no margin effects) with MAF=0.25

The aim of our simulation study was to evaluate the performance of different estimators not only in commonly assumed interaction models but also in more unusual interaction settings. We therefore selected five interaction models, of which the first four models were proposed by Wan et al. [26], the fifth model was based on Ritchie et al. [27] (there model 4) and was generated using the epistasis model discovery method of Moore et al. [28]. Of note, the models display interaction effects but little or no main effects and can be written as 3×3 contingency tables of odds for the first interacting variant with genotypes aa, aA, and AA, and the second interacting variant with genotypes bb, bB, BB, where the minor alleles are denoted by capital letters (see Supplement). The specific values are each determined by a prevalence parameter *α* and a multiplicative interaction parameter *θ* and are shown in Table 1 and visualized in the accompanying Figs. 1, 2, 3, 4 and 5 (after conversion to the case-control scenario).

**Table 1:** Odds tables and ideal HLO-matrices for five interaction models

	Odds			HLO		
Multiplicative model	bb	bB	BB	bb	bB	BB
aa	*α*	*α*	*α*	H	L	L
aA	*α*	*α*(1+*θ*)	*α*(1+*θ*)^2^	L	H	H
AA	*α*	*α*(1+*θ*)^2^	*α*(1+*θ*)^4^	L	H	H
Epistasis model	bb	bB	BB			
aa	*α*	*α*(1+*θ*)	*α*(1+*θ*)	L	H	H
aA	*α*(1+*θ*)	*α*	*α*	H	L	L
AA	*α*(1+*θ*)	*α*	*α*	H	L	L
Two allele interaction-model	bb	bB	BB	bb	bB	BB
aa	*α*	*α*	*α*(1+*θ*)	L	L	H
aA	*α*	*α*(1+*θ*)	*α*	L	H	L
AA	*α*(1+*θ*)	*α*	*α*	H	L	L
XOR model	bb	bB	BB	bb	bB	BB
aa	*α*	*α*(1+*θ*)	*α*	L	H	L
aA	*α*(1+*θ*)	*α*	*α*(1+*θ*)	H	L	H
AA	*α*	*α*(1+*θ*)	*α*	L	H	L
No margin-model with MAF=0.25	bb	bB	BB	bb	bB	BB
aa	0.03	0.10	0.08	L	H	H
aA	0.09	0.01	0.04	H	L	L
AA	0.10	0.01	0.00	H	L	O

*α*, prevalence parameter, and *θ*, multiplicative interaction parameter. High (H), low (L), and undetermined (O) risk genotype combinations in the interaction models

Three models (epistasis model, two allele interaction-model and XOR model) display only two levels of risk, whereas the multiplicative and the no margin-model a more complicated risk pattern. Specifically, the no margin-model was selected because it exhibits interaction effects in the absence of any main effects. Furthermore, all marginal odds are equal for this special model.

### Data simulation

We simulated data according to the five genotype interaction models using the software GAMETES [29, 30]. Genotypes were generated according to Hardy-Weinberg proportions, and a range of allele frequencies and patterns of risk-genotype associations was chosen [31].

For the models according to Wan (multiplicative model, epistasis model, two allele interaction-model and XOR model), three different minor allele frequencies (MAFs) were chosen (0.1, 0.2, and 0.4) leading to twelve odds tables (see Supplement). Setting the prevalence to 0.1 and the heritability to 0.03 for the multiplicative model and to 0.02 for the other models, the prevalence parameter *g* and the interaction parameter *t* were determined. The heritability is defined as described by Wan et al. [26]:

13 $$\begin{array}{@{}rcl@{}} h^{2} &=& \sum_{i,j=0}^{2} P(G_{ij}) \left (\frac{(P(D_{1}|G_{ij}) - P(D_{1}))^{2} }{P(D_{0})P(D_{1})} \right) \end{array} $$

For utilization in GAMETES, these odds tables were converted to penetrance tables. The no margin-model is directly taken from Ritchie et al. [27, 32]. With the MAF for the interacting variants set to 0.25 this model shows no marginal effects.

Each model was simulated with 100 variants, with 2 of them interacting, 1000 replicates (datasets), and 800 cases and 800 controls. The MAFs of the non-interacting SNPs were chosen randomly between 0.05 and 0.5.

For simulations under the null hypothesis, we used the four different interaction models according to Wan et al. [26] with three different MAFs, resulting in 12 models that were simulated with 1000 replicates each. Given that for every model, 100 SNPs were simulated with two interacting SNPs, we had 4949 non-interacting SNP pairs per model. Thus, a total of more than 50 million SNP pairs without interactions were simulated. Simulated data was evaluated at significance thresholds of 5×10^−2^, 5×10^−3^, 5×10^−4^, 5×10^−5^, and 5×10^−6^.

In the resulting data sets, we estimated $\widehat {IGmod}_{0}$ as described above.

### Comparison with other approaches

To compare our estimator with previous established approaches, we estimated logistic regression models testing for interaction in an additive genetic model with 1 df as implemented in the module epistasis of the software PLINK [11]. Furthermore, we performed likelihood ratio tests (LRT) comparing a model with 4 parameters (2 additive and 2 dominant terms) with the full model with 8 parameters [33]. Finally, another entropy-based approach with the test statistic *T*_*IG*_ was used as described by Fan et al. [21].

Essentially, Fan et al. [21] subtract the mutual information of two genetic variants estimated in the cases from the same quantities estimated in the controls.

### Submodel classification

To illustrate the underlying genetic models of the simulated data, we utilized one step of the model-based multifactor dimensionality reduction (MB-MDR) algorithm, which is an efficient algorithm to perform multiple testing in epistasis screening [34]. The procedure tabulates the frequencies of cases and controls in the 3×3 genotype combinations and uses a test for association between the trait and the specific genotype combination. The test is performed for every cell of the 3×3 contingency table and denotes the individuals with the genotype combination of the specific cell as having a high risk of being affected (H), or a low risk (L), or not sufficient evidence or information (O). The result is a 3×3 matrix, denoted as HLO-matrix. For illustration, these matrices are shown in Table 1 (right) under the assumption that sample size is large enough to yield sufficient evidence.

### Real data

We use the data from the genome-wide association study by the North American Rheumatoid Arthritis Consortium (NARAC) that were also analyzed by Liu et al. [2]. The data set comprises genotype data of 2,062 individuals, 868 cases with RA and 1,194 controls, predominantly of Northern European origin. The data had been genotyped on the Illumina 550k platform. After exclusion of monomorphic SNPs and SNPs showing deviation from HWE at *p*<0.0001, 515,680 SNPs were available for further analysis [24]. Quality control procedures included removing individuals who had a low overall call rate (<95 %) of SNPs [23]. From these data we select the HLA-region on chromosome 6 encompassing 2010 SNPs. This area offers a large number of SNPs of which many are associated with RA, and previous analyses hinted at gene-gene interactions in this region. To reduce the number of SNP-pairs to investigate, we further selected only SNPs overlapping with the 749 SNPs analyzed by Liu et al. [2]. Because of the assumption of no LD, we furthermore eliminated all SNP pairs with a LD of *r*^2^>0.01.

### Significance thresholds for the modified IGENT interaction evaluation

As described above, the estimator $\widehat {IGmod}_{0}$ asymptotically and approximately follows a gamma distribution with shape parameter 4 and scale parameter $\frac { 1}{N \ln (2)}$ under the null hypothesis of conditional independence of the genetic variants [25].

Because of the fact that

- the characteristic of the underlying distribution is given only asymptotically and approximately,
- the Bonferroni correction is very conservative, and
- the test statistic is dependent on allele frequencies and marginal effects,

fixing the shape parameter at 4 is partially very conservative. Thus, we utilize alternative cut-offs to identify relevant pairs of interacting SNPs. For the simulated data, we set the global significance level to *α*=0.05 and apply a Bonferroni correction to adjust for the number of interactions being tested from the gamma distribution with shape parameter 2 (liberal criterion). For the real data, we also set the global significance level to *α*=0.05 but apply a Bonferroni correction to adjust for the number of interactions being tested from the gamma distribution with shape parameter 4 (conservative criterion).

Assuming the scale parameter of $\frac { 1}{N \ln (2)}$, this leads to a cut-off at <0.012836 (see below) for the simulated data and at <0.017331 for the real data.

For the regression analysis we set the global significance level to *α*=0.05 with Bonferroni correction (for the simulated data based on the number of SNP pairs), which leads to significance levels of 1×10^−5^ for the simulated data and 5×10^−8^ for the real data.

## Results

### Evaluation of type I error

Firstly, the type I error is evaluated in the simulation data. For the null simulation we took about 59 million SNP pairs like described above. The global significance level of 5% is controlled using the liberal cut-off of 0.012836 (Table 2). This is due to the structure of the null simulation data with different MAFs, but only a small portion of SNPs with main effects. Table 2 shows that the type I error matches the different thresholds of 5×10^−2^, 5×10^−3^, 5×10^−4^, 5×10^−5^, and 5×10^−6^ under the gamma distribution with shape parameter 2 very well.

**Table 2:** Type I error in simulated data at cut-off from gamma distribution with shape parameter 2

Threshold	Type I error
5×10^−2^	5.38×10^−2^
5×10^−3^	5.39×10^−3^
5×10^−4^	5.41×10^−4^
5×10^−5^	5.36×10^−5^
5×10^−6^	5.25×10^−6^

### Evaluation of power

**Fig. 6:**
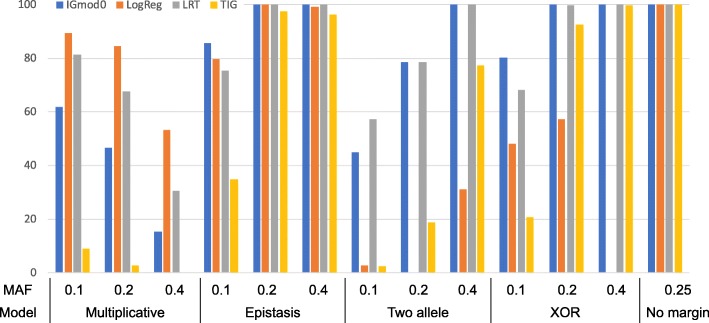
Power in simulation data. Approaches are the proposed estimator IGmod0 (with cutoff = 0.012836), logistic regression (LogReg), likelihood ratio test (LRT), and TIG as proposed by Fan et al. [21] for different minor allele frequencies (MAF)

Figure 6 shows the power to detect the interaction in the simulated data for all interaction models and MAF settings. There are four scenarios that display a limited power of <80%, i.e., the multiplicative model with MAF=0.1, 0.2 and 0.4, and the two allele interaction-model with MAF=0.1. By comparison, the logistic regression model shows overall lower power to detect the interactions, with scenarios having power <80% including the multiplicative model with MAF=0.4, the epistasis model with MAF=0.1, the two allele interaction-model as well as the XOR model with all MAFs. The no margin-model provides satisfying power for both estimators, the proposed estimator and logistic regression. Thus, specifically for the epistasis model at a low MAF and unconventional models at all MAFs, the proposed estimator $\widehat {IGmod}_{0}$ performs better than the classical logistic regression in detecting interactions. The LRT shows overall comparable power as $\widehat {IGmod}_{0}$ with better performance in the multiplicative model but lower power in the epistasis and XOR models with low MAF. Finally, the estimator *T*_*IG*_ provides good results (power >80%) in the epistasis and XOR models with higher MAFs and for the no margin-model, but the power is lower than that of the proposed estimator throughout.

### Submodel classification

We observed an intermediate power of $\widehat {IGmod}_{0}$ depending on the specific setting and interaction model. Thus, to get a more detailed impression of the underlying interactions, we classified the frequently occuring identified interaction pairs using HLO-matrices as described above.

#### Multiplicative model

Table 3 shows the frequencies of the more common submodels occuring in the simulation of the multiplicative model with MAF=0.2 along with the power of $\widehat {IGmod}_{0}$ and the logistic regression to detect the interaction.

**Table 3:** HLO-matrix for frequent submodels in the multiplicative model (MAF=0.2) with frequency and power in simulated data

					Power	Power
	HLO-matrix			Freq	$\widehat {IGmod }_{0}$	Logistic regression
	bb	bB	BB	0.07	0.90	1
aa	H	L	L			
aA	L	H	H			
AA	L	H	H			
	bb	bB	BB	0.36	0.76	0.99
aa	H	L	L			
aA	L	H	H			
AA	L	H	O			
	bb	bB	BB	0.08	0.53	0.88
aa	H	L	L			
aA	L	H	H			
AA	L	O	O			
	bb	bB	BB	0.07	0.49	0.91
aa	H	L	L			
aA	L	H	O			
AA	L	H	O			
	bb	bB	BB	0.07	0.42	0.83
aa	H	L	L			
aA	L	O	H			
AA	L	H	O			

Frequency (freq) of specific submodels and power to detect the interaction for specific submodels. Submodels are described by HLO-matrices as illustrated in Table 1

It can be seen that $\widehat {IGmod}_{0}$ provides high power for mostly complete submodels, in which almost every cell contains enough information to perform the association test. In incomplete submodels, in which not all cells of the HLO-matrix show an effect or the cells contain not enough information to perform the association test, $\widehat {IGmod}_{0}$ shows more difficulty to detect the interaction. In contrast, the regression model provides a power higher than 80% in all submodels.

#### Epistasis model

Analogously, Table 4 shows the respective submodel results for the epistasis model with MAF=0.1.

**Table 4:** HLO-matrix for frequent submodels in the epistasis model (MAF=0.1) with frequency and power in simulated data

					Power	Power
	HLO-matrix			Freq	$\widehat {IGmod }_{0}$	Logistic regression
	bb	bB	BB	0.14	0.99	0.98
aa	L	H	H			
aA	H	L	O			
AA	H	O	O			
	bb	bB	BB	0.22	0.99	0.87
aa	L	H	O			
aA	H	L	O			
AA	H	O	O			
	bb	bB	BB	0.23	0.96	0.91
aa	L	H	H			
aA	H	L	O			
AA	O	O	O			
	bb	bB	BB	0.41	0.95	0.65
aa	L	H	O			
aA	H	L	O			
AA	O	O	O			

Frequency (freq) of specific submodels and power to detect the interaction for specific submodels. Submodels are described by HLO-matrices as illustrated in Table 1

Notably, many results with incomplete submodels were obtained in which one or more cells of the HLO-matrix did not show evidence for association or did not contain enough information for association testing. For example, 41% of the relevant pairs led to HLO-matrices which show association only in four out of nine possible cells. However, $\widehat {IGmod}_{0}$ provides high power for all submodels, whereas the regression model has more difficulty detecting interactions in incomplete submodels.

### Real data

In the analysis of the real data set, we obtain 211 relevant SNP pairs, of which 102 display a model similar to the multiplicative model. Further 90 relevant SNP pairs resemble the epistasis model, and 19 SNP pairs follow more unconventional models. Compared to the regression analysis, our estimator identifies 31 SNP pairs as relevant that are not detected by the regression approach, and these are shown in Tables 5 and 6.

**Table 5:** Interactions detected by $\widehat {IGmod}_{0}$ but not logistic regression

SNP pair	pos SNP 1	pos SNP 2	Submodel	MAF 1	MAF 2	p SNP 1	p SNP 2	TS pair	*p* value
rs1063355:rs7774434	32735692	32765556	Multi	0.37	0.44	9.68E-14	5.09E-19	0.018961	6.31E-09
rs1063355:rs9275374	32735692	32776504	Multi	0.37	0.35	9.68E-14	1.22E-56	0.020315	1.11E-09
rs1063355:rs9275388	32735692	32777062	Multi	0.37	0.34	9.68E-14	2.54E-53	0.018774	8.01E-09
rs1063355:rs9275390	32735692	32777134	Multi	0.37	0.35	9.68E-14	1.22E-56	0.020315	1.11E-09
rs1063355:rs9275393	32735692	32777417	Multi	0.37	0.35	9.68E-14	1.47E-56	0.020343	1.07E-09
rs1063355:rs9275406	32735692	32777933	Multi	0.37	0.34	9.68E-14	2.29E-56	0.020699	6.78E-10
rs1063355:rs9275407	32735692	32778015	Multi	0.37	0.34	9.68E-14	1.44E-53	0.021665	1.95E-10
rs1063355:rs9275418	32735692	32778222	Multi	0.37	0.35	9.68E-14	7.88E-57	0.020276	1.17E-09
rs1063355:rs9275424	32735692	32778554	Multi	0.37	0.35	9.68E-14	7.88E-57	0.020314	1.11E-09
rs1063355:rs9275425	32735692	32778852	Multi	0.37	0.34	9.68E-14	1.96E-52	0.019848	2.03E-09
rs1063355:rs9275427	32735692	32778893	Multi	0.37	0.35	9.68E-14	6.95E-57	0.020023	1.62E-09
rs1063355:rs9275428	32735692	32778956	Multi	0.37	0.35	9.68E-14	3.13E-56	0.019946	1.79E-09
rs1063355:rs9275439	32735692	32779499	Multi	0.37	0.34	9.68E-14	6.63E-54	0.020084	1.50E-09
rs2256175:rs9275224	31488428	32767856	Multi incompl	0.44	0.37	3.24E-15	6.52E-90	0.018158	1.76E-08
rs1055569:rs4424066	31548061	32462406	Multi incompl	0.32	0.45	3.23E-08	1.07E-66	0.018868	7.11E-09
rs1055569:rs3817973	31548061	32469089	Multi incompl	0.32	0.45	3.23E-08	1.91E-67	0.019307	4.06E-09
rs1055569:rs2076530	31548061	32471794	Multi incompl	0.32	0.45	3.23E-08	4.92E-64	0.018858	7.20E-09
rs9267911:rs3130320	32313088	32331236	Multi incompl	0.41	0.28	3.26E-36	2.10E-32	0.020101	1.46E-09
rs1055569:rs2395157	31548061	32456123	Epi	0.32	0.38	3.23E-08	2.27E-60	0.017474	4.18E-08
rs2844509:rs3817963	31618903	32476065	Epi	0.21	0.40	9.59E-19	7.20E-58	0.018005	2.13E-08
rs6941112:rs9275595	32054593	32789333	Epi	0.37	0.32	6.08E-16	9.50E-63	0.017436	4.38E-08
rs9268615:rs6903608	32510867	32536263	Epi	0.47	0.23	6.58E-44	8.39E-53	0.031001	8.87E-16
rs2395185:rs7745656	32541145	32788948	Epi	0.43	0.22	6.41E-71	1.71E-38	0.019093	5.33E-09
rs477515:rs7745656	32677669	32788948	Epi	0.42	0.22	6.18E-67	1.71E-38	0.026765	2.46E-13
rs2516049:rs7745656	32678378	32788948	Epi	0.42	0.22	1.83E-66	1.71E-38	0.026215	5.08E-13
rs382259:rs2647012	32317005	32772436	XOR	0.22	0.29	6.52E-28	2.26E-54	0.017355	4.86E-08
rs382259:rs2856717	32317005	32778286	XOR	0.22	0.29	6.52E-28	5.05E-56	0.017723	3.05E-08
rs382259:rs2858305	32317005	32778442	XOR	0.22	0.29	6.52E-28	5.05E-56	0.017723	3.05E-08
rs382259:rs9275572	32317005	32786977	XOR	0.22	0.31	6.52E-28	2.86E-59	0.018208	1.65E-08
rs412657:rs405875	32319063	32323166	Other	0.35	0.50	2.94E-42	5.21E-21	0.017883	2.49E-08
rs412657:rs3115573	32319063	32326821	Other	0.35	0.50	2.94E-42	2.44E-20	0.017684	3.20E-08

Position (pos) of SNPs as base pairs on chromosome 6, submodel of interaction as detailed in Table 6, minor allele frequencies (MAF), p SNP 1 and p SNP 2 as *p*-values from 1st order calculation, TS pair as value of the test statistic from 2nd order calculation, *p*-value as result from 2nd order calculation

**Table 6:** Submodel categories for interactions detected by $\widehat {IGmod}_{0}$ but not logistic regression

Submodel	HLO-matrix		
Multi	bb	bB	BB
aa	H	L	L or O
aA	L	H	H
AA	L	H or O	H or O
Multi incompl	bb	bB	BB
aa	H	L or O	L or O
aA	L or O	O	H or O
AA	L or O	H or O	H or O
Epi	bb	bB	BB
aa	L	H	H or O
aA	H or O	L or O	O
AA	H or O	L or O	L or O
XOR	bb	bB	BB
aa	O	O	L
aA	O	O	H
AA	L	H	O
Other	bb	bB	BB
aa	L or O	O	H or O
aA	O	O	H or O
AA	H	H	L

HLO-matrices for the submodels of interaction in Table 6

Most of the interaction pairs essentially follow a multiplicative model (multi) or an incomplete multiplicative model (multi incompl). There are seven SNP pairs for which the model resembles the epistatic model (epi), and the remaining are either similar to the XOR model or are difficult to classify (other).

Of note, 39 SNP pairs were detected by the regression approach at a significance level of 1×10^−9^ but not by $\widehat {IGmod}_{0}$. All of these pairs belong to the categories of multiplicative and epistatic models. They do achieve a relatively high test statistic value of more than 0.01, but do not meet our conservative significance criterion of 0.017331.

Comparing the SNP pairs identified by $\widehat {IGmod}_{0}$ with the results by Liu et al. [2], we find that all eight SNP pairs reported by Liu et al. [2] are also detected by our proposed estimator (Table 7). Notably, all of these eight pairs belong to either the multiplicative or the epistasis model and have relatively high MAFs (>32*%*).

**Table 7:** Results for interactions reported by Liu et al. [2]

SNP pair	p SNP 1	Gene 1	p SNP 2	Gene 2	Submodel	LD	TS pair	*p* value
rs9275595:rs10807113	9.50E-63	DQA2 (F5U)	1.30E-05	DQB2 (F3U)	Multi	0.0026	0.03843	4.08E-20
rs9275390:rs10807113	1.22E-56	DQA2 (F5U)	1.30E-05	DQB2 (F3U)	Multi	0.0001	0.039163	1.51E-20
rs9275390:rs2051549	1.22E-56	DQA2 (F5U)	4.96E-01	DQB2 (Intron)	Multi	0.0074	0.029437	7.13E-15
rs2858332:rs10807113	2.90E-06	DQA2 (F5U)	1.30E-05	DQB2 (F3U)	Epi	0.0082	0.043533	4.01E-23
rs7774434:rs10807113	5.09E-19	DQA1 (F3U)	1.30E-05	DQB2 (F3U)	Multi	0.0035	0.029443	7.07E-15
rs7774434:rs2051549	5.09E-19	DQA1 (F3U)	4.96E-01	DQB2 (Intron)	Multi	0.0011	0.042317	2.10E-22
rs9275390:rs6901084	1.22E-56	DQA2 (F5U)	1.39E-02	DQB2 (F5U)	Epi	0.0088	0.025216	1.89E-12
rs2858332:rs6901084	2.90E-06	DQA2 (F5U)	1.39E-02	DQB2 (F5U)	Multi	0.0091	0.043533	2.90E-21

Submodel of interaction as detailed in Table 6, p SNP 1 and p SNP 2 as *p*-values from 1st order calculation,

linkage disequilibrium (LD) between SNPs, TS pair as value of the test statistic from 2nd order calculation,

*p*-value as result from 2nd order calculation

Finally, we compared our results with those by Chattopadhyay et al. [3]. Their 20 top ranked 2-way interactions within the HLA region contain SNPs with a low MAF, but they were excluded from our evaluation either due to an LD of more than 0.01 or because they were not contained in the set of 749 SNPs analyzed by Liu et al. [2].

## Discussion

In this paper we proposed a modification of the IGENT estimator for second order genetic interactions, which exploits the advantages of the entropy methods, but controls the type I error. It is a tool designed specifically to detect various patterns conditional on a baseline model, and foremost to detect interactions. The estimator asymptotically and approximately follows a gamma distribution with a shape parameter that depends on the existence of margin effects. The calculation can be made within the IGENT software, which is simple to apply and very fast.

Our simulations show that the power of our method depends on marginal effects, and the lowest power was observed for the multiplicative model. Instead, the advantages of our modification lie in the possibility to detect interactions for unconventional interaction models (like two allele interaction-model and XOR model), and for incomplete models (e.g. like last HLO-matrix in Table 4), in which not all genotype combinations are observed with sufficient frequency. Thus, $\widehat {IGmod}_{0}$ outperforms all other approaches in the XOR and epistasis models especially at low MAFs and is better than all approaches except the LRT in the two allele model, where $\widehat {IGmod}_{0}$ and the LRT are comparable.

In the analysis of the real data on RA, we find the results of the simulation confirmed. We observe a similar number of SNP pairs detected by one but not the other approach. Again, our modification is more likely to uncover unconventional or incomplete models.

In general, our results confirm the conclusion of Zuo et al. [20] that GenoCMI measures achieve a control of the false positive error in the presence of main effects.

## Conclusions

In conclusion, we proposed a modification of the IGENT method, which is a fast and efficient entropy-based interaction analysis algorithm [22]. The modification reduces the type I error, so it can easily identify second order gene-gene interactions on a genome-wide scale. The analysis of simulated and real data has shown that, in contrast to classical regression approaches, more unconventional interaction models can be detected with this approach, which makes it an attractive complement to established analysis methods.

## Supplementary information

**Additional file 1** Odds table for 13 settings. Values of odds for 4 models with 3 different minor allele frequencies (MAFs) (0.1, 0.2, 0.4 resp. for the interacting SNPs (genotypes)) and 1 model with MAF = 0.25. The minor alleles are denoted by capital letters.

## Data Availability

Not applicable

## Abbreviations

ANOVA: Analysis of Variance Technique
CMI: Conditional Mutual Information
GAMETES: Genetic Architecture Model Emulator for Testing and Evaluating Software
GenoCMI: Special Measure for CMI
GWAS: Genome-wide association study
HLA: Human Leukocyte Antigens
HLO: High, Low or No Evidence
HWE: Hardy-Weinberg Disequilibrium
IG: Information Gain
IGENT: Interactions analysis method in Genome-wide scale based on ENTtropy
LD: Linkage Disequilibrium
MAF: Minor Allele Frequency
MB-MDR: Model-Based Multifactor Dimensionality Reduction
MDR: Multifactor Dimensionality Reduction Method
NARAC: North American Rheumatoid Arthritis Consortium
PLINK: Tool Set for Whole-Genome Association and Population-Based Linkage Analyses
RA: Rheumatoid Arthritis
RF: Random Forests Method
SNP: Single-Nucleotide Polymorphism
SVM: Support Vector Machine Method
XOR: eXclusive OR Logic Disjunction
