# Supplementary material for: Modified entropy-based procedure detects gene-gene-interactions in unconventional genetic models
Source: BMC Med Genomics. 2020 Apr 23;13:65. doi: 10.1186/s12920-020-0703-4 (PMC7181579; doi:10.1186/s12920-020-0703-4)
Supplement: Supplementary file 1 — Additional file 1 Odds table for 13 settings. Values of odds for 4 models with 3 different minor allele frequencies (MAFs) (0.1, 0.2, 0.4 resp. for the interacting SNPs (genotypes)) and 1 model with MAF = 0.25. The minor alleles are denoted by capital letters. [file 12920_2020_703_MOESM1_ESM.pdf]

Odds contingency table with margin odds

	Odds			
Setting 1 (model 1 with MAF=0.1)	bb	bB	BB	margin
aa	0.10	0.10	0.10	0.10
aA	0.10	0.44	1.98	0.18
AA	0.10	1.98	39.22	0.83
margin	0.10	0.18	0.83	
Setting 2 (model 1 with MAF=0.2)	bb	bB	BB	margin
aa	0.09	0.09	0.09	0.09
aA	0.09	0.21	0.48	0.14
AA	0.09	0.48	2.55	0.31
margin	0.09	0.14	0.31	
Setting 3 (model 1 with MAF=0.4)	bb	bB	BB	margin
aa	0.07	0.07	0.07	0.07
aA	0.07	0.12	0.20	0.12
AA	0.07	0.20	0.52	0.20
margin	0.07	0.12	0.20	
Setting 4 (model 2 with MAF=0.1)	bb	bB	BB	margin
aa	0.08	0.20	0.20	0.10
aA	0.20	0.08	0.08	0.17
AA	0.20	0.08	0.08	0.17
margin	0.10	0.17	0.17	
Setting 5 (model 2 with MAF=0.2)	bb	bB	BB	margin
aa	0.06	0.17	0.17	0.10
aA	0.17	0.06	0.06	0.13
AA	0.17	0.06	0.06	0.13
margin	0.10	0.13	0.13	
Setting 6 (model 2 with MAF=0.4)	bb	bB	BB	margin
aa	0.06	0.17	0.17	0.13
aA	0.17	0.06	0.06	0.10
AA	0.17	0.06	0.06	0.10
margin	0.13	0.10	0.10	
Setting 7 (model 3 with MAF=0.1)	bb	bB	BB	margin
aa	0.10	0.10	0.40	0.10
aA	0.10	0.40	0.10	0.15
AA	0.40	0.10	0.10	0.35
margin	0.10	0.15	0.35	
Setting 8 (model 3 with MAF=0.2)	bb	bB	BB	margin
aa	0.09	0.09	0.25	0.10
aA	0.09	0.25	0.09	0.14
AA	0.25	0.09	0.09	0.19
margin	0.10	0.14	0.19	
Setting 9 (model 3 with MAF=0.4)	bb	bB	BB	margin
aa	0.07	0.07	0.19	0.10
aA	0.07	0.19	0.07	0.13
AA	0.19	0.07	0.07	0.12
margin	0.10	0.13	0.12	
Setting 10 (model 4 with MAF=0.1)	bb	bB	BB	margin
aa	0.08	0.20	0.08	0.10
aA	0.20	0.08	0.20	0.18
AA	0.08	0.20	0.08	0.10
margin	0.10	0.18	0.10	
Setting 11 (model 4 with MAF=0.2)	bb	bB	BB	margin
aa	0.07	0.17	0.07	0.10
aA	0.17	0.07	0.17	0.14
AA	0.07	0.17	0.07	0.10
margin	0.10	0.14	0.10	
Setting 12 (model 4 with MAF=0.4)	bb	bB	BB	margin
aa	0.06	0.17	0.06	0.11
aA	0.17	0.06	0.17	0.12
AA	0.06	0.17	0.06	0.11
margin	0.11	0.12	0.11	
Setting 13 (model 5 with MAF=0.25)	bb	bB	BB	margin
aa	0.03	0.10	0.08	0.06
aA	0.09	0.01	0.04	0.06
AA	0.10	0.01	0.00	0.06
margin	0.06	0.06	0.06	
